# Structural validity and reliability of the “Oral Health Assessment Tool” applied by speech-language therapists in a population of older Chilean people

**DOI:** 10.1186/s12903-023-02725-5

**Published:** 2023-01-17

**Authors:** Camilo Morales, Felipe Henríquez, Sergio Muñoz

**Affiliations:** 1 Departamento de Procesos Terapéuticos, Facultad de Ciencias de La Salud, Universidad Católica de Temuco, Manuel Montt No. 056, Campus San Francisco, edificio B, Temuco, Chile; 2 Departamento de Salud Pública, Facultad de Medicina-CIGES, Universidad de Frontera, Temuco, Chile

**Keywords:** Older people, Oral health, Validity, Reliability, Validation, Speech-language therapist, Chile

## Abstract

**Background:**

A good state of oral health allows people to communicate and eat. This topic is relevant in older people given its close relationship with their general health. At present, health challenges are directed at detecting and preventing oral disorders and are seen to exclusively by dentists, because the existing instruments can only be applied by them. However, speech-language therapists undergo similar training, which would allow them to collaborate in these processes. In this context, the Oral Health Assessment Tool (OHAT) is a detection instrument with good psychometric properties that is currently available for non-dental use. The objective of this study is the translation into Chilean Spanish of the OHAT and a structural validation of that version for application by these professionals.

**Materials and methods:**

A mixed qualitative-quantitative study was carried out. The OHAT instrument was adapted to Chilean Spanish and subsequently subject to structural validity and evaluation of internal consistency reliability, as well as a valuation of its reproducibility in 286 older people (166 female, 120 male) from different health contexts.

**Results:**

The cultural adaptation of the instrument proved to be semantically consistent with the original instrument. Its application was considered to be speedy and simple in the pre-test. The confirmatory factor analysis evidenced the unidimensionality of the OHAT. In addition, the instrument shows good internal consistency and test–retest reliability.

**Conclusions:**

The OHAT instrument was considered to possess adequate structural validity and test–retest reliability properties. Its usefulness in the context of oral health disorders of this population in Chile is discussed.

## Background

Oral health is defined as the ability to speak, smile, smell, taste, touch, chew and swallow, as well as to transmit emotions through facial expressions with confidence, without pain, discomfort and/or craniofacial disorders [1]. It allows people to communicate and feed themselves effectively [2]. This construct is particularly important given its implication and close relationship with overall health [3]; for this reason, poor oral health (usually expressed by the presence of caries, periodontal diseases, oral pain or cancer) affects the self-perception of a person both in terms of self-esteem and self-confidence [4].

The progressive understanding of the consequences associated with this construct has given rise to the implementation of oral health promotion plans and programs at a local and international level. At the end of the twentieth century, modest reductions in the prevalence of dental caries were achieved in children [5, 6]; however, focus on older people is still incipient. Considering current demographic changes at a global level, promotion and prevention efforts vis-à-vis this latter group has become increasingly relevant. As people reach older age, their needs, including oral health issues, require continual attention. The aging process and associated changes affecting the population pose major challenges to maintain an optimal state of health throughout the lives of individuals and populations [5, 7]. In this context, several research efforts have sounded the alarm regarding the risks that poor oral health and mouth diseases have on general health, particularly in older people [3, 8, 9]. The literature describes a link between oral health and systemic diseases. For example, an association between the number of missing teeth with heart disease has been reported; periodontal disorders have been related to cardiovascular disease, atherosclerosis, subclinical lower artery disease, strokes, metabolic and lipid disorders and obesity [3, 10]. Additionally, pathologies such as diabetes and respiratory ailments can be related to poor oral health conditions [11]; Chalmers [10], reported that state of dental health, loss of teeth and temporomandibular disorders are associated with auditory impairment. These various relationships acquire greater relevance because older people seem prone to present oral health problems [12]. The data indicate that the elderly population tends to have poor oral health largely due to dental care deficiencies during their entire lifetime. Elderly people with some degree of dependence or limited autonomy tend to present worse oral conditions [13]. Therefore, the risk of developing these problems in older people with attention needs is high, particularly for those with severe dependence problems living in nursing homes or who are hospitalized. This risk is also related to social patterns in the older population, such as income level, knowledge regarding oral health care or access to health facilities [14], and therefore its prevalence varies depending on these variables. Although several studies have tried to look into and establish oral health intervention programs for older people, this is still described as insufficient or jeopardized [3, 14].

Although the detection of alterations or their oversight are carried out through clinical examinations performed by dentists, these methods are increasingly more difficult to use due to the high cost and scarcity of human resources, even in high-income countries [4, 15]. Therefore, alternative and less resource-demanding approaches are needed. In this context, it is important to mention the existence of self-report questionnaires, associated mainly with the oral health dimension in relation to quality of life. However, because it is based on the ability of a person to report any adverse dental symptom, it increases the risk of bias, especially in people with some kind of cognitive impairment [16]. Moreover, the majority of clinical instruments or oral health indices are designed to be used by dentists and dental hygienists, but they are not suitable for use by non-dental professionals [17, 18], even though, given their disciplinary similarity, speech-language therapists would be suitable for this purpose. Therefore, the availability of valid and reliable instruments enabling the evaluation of oral health through the observation of structures by trained professionals would be especially relevant.

In this sense, at an international level there are tools available to evaluate and detect oral health problems, such as the Oral Health Assessment developed by the World Health Organization (WHO) [19] in its version for adults; the Geriatric Oral Health Assessment Index (GOHAI) [20] specifically targeted at older people, and the Oral Health Impact Profile (OHIP) [5]. However, these last two measure the perception of individuals regarding their own oral health [21], with their respective limitations.

Conversely, reports in the literature point to the Oral Health Assessment Tool (OHAT) as an instrument that measures oral health intended as an interdisciplinary valuation of this condition. That is to say, its application by other professionals, specifically nurses and speech-language therapists has been signaled as feasible [17, 18], making it possible for this tool not to be linked solely to dentists. The OHAT consists of eight categories aimed at identifying oral health impairments as well as pinpointing the need for prevention actions or referrals for dental intervention, making it a useful instrument for the detection of possible disorders and their early management in elderly adults, whether or not dependent. On the basis of the original (Australian) instrument, validation studies of similar instruments have been conducted in Germany, Japan, Brazil, Indonesia, the Netherlands and Turkey [22–27]. At present, the OHAT has not been translated or validated in the Spanish language in any country, including Chile.

Given the relevance and usefulness that this instrument represents for health policies and clinical approaches regarding oral health of the older adult population in different contexts, the objective of this study is to determine the structural validity and reliability of the OHAT instrument in the Chilean older adult population. We hypothesize the OHAT correctly adjust to a one-factor model, consistent with the “Disease and condition status” sub-dimension of the oral health framework proposed by the World Dental Federation (FDI), defined as the threshold of severity or level of progression of a possible oral health pathology, which also includes pain or discomfort [1]. Moreover, we hypothesize that the instrument has adequate internal consistency and test–retest reliability.

## Materials and methods

### Study design

This study has a mixed qualitative-quantitative design, using a methodological approach to validating a measurement instrument. Qualitative, because it aims to establish the cultural validation of the OHAT questionnaire, contemplating phases of translation and evaluation of the coverage and façade of the scale. The quantitative approach is analytical and relational.

### Recruitment of participants

The participants were people aged 60 years or more, proceeding from residences or groups for the elderly, health care groups for prostrated and hospitalized patients from different institutions in the La Araucanía region of Chile, during years 2019 and 2020. Excluded from the study were older people presenting difficulties regarding responsiveness or the ability to follow simple instructions in the context of the application of the instrument, given a situation of severe dependence, dementia or impaired level of consciousness, which were determined via clinical records, the application of the Barthel Index and the Pfeiffer Short Portable Mental State Questionnaire (SPMSQ), respectively.

### Instruments

- For the sociodemographic characterization and subsequent descriptive statistical analysis, a general information record of the participants was drawn up, which included gender, age, diagnosis of pathologies such as dementia, diabetes or hypertension, level of dependence and type of nutrition, among others.
- *Barthel Index* used to measure the level of dependency of the participants with the objective of complementing the characterization of the population under study. It assesses a person's ability to perform 10 activities of daily living, for example: eating, personal hygiene, going up and down stairs, among others. The total score ranges from 0 to 100 points, being classified within the following ranges: < 20 points: fully dependent; 20 to 40 points severely dependent, 45 to 55 points moderately dependent and 60 or more points mildly dependent [28].
- *Oral Health Assessment Tool (OHAT)* measurement instrument, which on this occasion was administered by a speech-language therapist. It is made up of eight items (lips, tongue, gums and tissues, saliva, natural teeth, dentures, oral cleanliness and dental pain), the answers to which are organized according to a Likert scale from 0 to 2 points, where 0 indicates absence of oral health issues (healthy) and 2 suggests possible disease (unhealthy). The score of each item reflects a description of the observed structures (healthy, signs of possible disease and unhealthy). For its application, a professional with competence in the discipline or formal training is required. Flashlight, gloves and mask should be used in the case of hospitalized patients or people with poor oral hygiene of orofacial structures. The instrument can also be applied to persons with cognitive alterations but who are responsive to simple instructions [18].

### Sample size

The estimation of the sample size required to achieve structural validity was conducted following the criteria proposed by Streiner et al. [29], consisting of 10 individuals participating per item of the measurement instrument, with a minimum of 200 persons when the number of items is small. Consequently, a non-probabilistic convenience sampling was implemented, with a total of 286 participants recruited.

The test–retest reliability estimation was carried out as proposed by Donner et al. [30], with an Intraclass Correlation Coefficient (ICC) of 0.6 as the acceptable minimum and 0.8 expected, a significance level of α = 0.05 and a power (1 − β) of 80%, for two measurements with a dropout rate of 10%. In this case, the calculated minimum sample was 49 persons. In this study, 76 persons were finally included for the purpose of this analysis.

### Procedure

This study was carried out in two phases. In the first place, the original version of the OHAT was translated into Chilean Spanish and adapted to its culture. In the second phase, its psychometric properties were assessed in a sample of older people. This study received the approval of the scientific ethics committee of the Universidad Católica de Temuco, under resolution No. 40/20. The participation of the subjects was completely voluntary or authorized by a family member or tutor, and they were at liberty to drop out of the study without this involving any detriment to the daily care provided in their respective facilities. Consequently, each participant or tutor signed an informed consent form, evidencing their free and voluntary participation in accordance with the principles of the Helsinki Decalogue [31].

#### Cultural adaptation

First of all, authorization to adapt the instrument was requested from the Iowa Geriatric Education Center by e-mail. The cultural adaptation consisted of the following [32]:

- *Direct translation* undertaken by two independent bilingual translators whose mother tongue is Chilean Spanish. The first addressed the study blind and the second was informed of its objective. In addition to translating, they identified comprehension and translation problems arising from semantic elements that were difficult to understand or confusing. After this, the translators got together to analyze their texts, detect discrepancies between them and produce the consensus version. They were also asked to maintain the conceptual equivalence of terms rather than a literal translation, when necessary.
- *Reverse translation* translation of the consensus version produced by the bilingual translators back to the original language. This was carried out by a bilingual speech-language therapist whose mother tongue is English. This person rated each of the translated items in terms of (1) Semantic/conceptual equivalence (maintaining most of the linguistic-semantic terms as expressed in the original translation); (2) Functional equivalence (grammatical modification of the original idea, maintaining the conceptual equivalence) and (3) Non-evident equivalence (major departure from the concept). Whenever any translated word or phrase fell into the third category, an alternative tending toward equivalence or a justification for the change was reached by consensus by the experts.
- *Consolidation and final production of the instrument* a committee was formed made up of the translators who generated the consensus version, a speech-language therapist trained in geronto-geriatrics and a dentist, who were presented the two initial versions provided by the translators, the consensus version and the reverse translation submitted by the speech-language therapist. Discrepancies regarding the translation of the instrument were discussed in terms of quality of translation, maintenance of the linguistic or functional equivalence and the modifications associated with contextual pertinence made to arrive at the final instrument.
- *Pre-test* three speech-language therapists applied the OHAT to a group of 30 persons. After this they were asked for feedback, to allow them to identify difficulties experienced with regard to understanding some item(s) of the instrument, or aspects related to the instructions, semantics, grammar or comprehension regarding the type of answer required.

#### Collection of data for structural validity

To carry out this procedure, the participants were first required to answer a brief questionnaire in order to collect information regarding their sociodemographic background. After this, the Short Portable Mental Questionnaire and the Barthel index were used to complement the general data. Once the base characteristics and eligibility of the subjects had been checked, the OHAT instrument was applied. Given the diversity of contexts, its application took place in a speech-language attention booth in the respective physical space of the participating centers or in the residences themselves, safeguarding the lighting conditions and absence of distractors, and the delivery of clear (protocolized) instructions by the evaluator, who was trained for this purpose.

#### Procedure for obtaining evidence of reliability

After the first application of the OHAT, the participants in the study were asked to answer it on a second occasion, within a maximum period of 7 days, to determine the test–retest reliability.

#### Statistical analysis

A sociodemographic characterization of the study population was carried out, using descriptive statistics, specifically central tendency measures and dispersion for quantitative variables, and absolute and relative frequencies for categorical variables.

To achieve the objective regarding the structural validity, a confirmatory factor analysis (CFA) [33] was carried out to corroborate the factor structure of the sub-dimension “Disease and condition status”, which is part of oral health as reported by the literature [1]. In this case, given that the instrument variables are ordinal in nature, the diagonally weighted least squares (DWLS) estimator was used, since the data are ordinal and do not meet the assumption of multivariate normality [34]. For model assessment, several model fit indices were calculated, including the comparative fit index (CFI), the Tucker-Lewis index (TLI), the root mean square error of approximation (RMSEA), in addition to the standardized root mean square error of approximation (SRMSEA). For CFI and TLI, values greater than or equal to 0.95 were considered acceptable [35]. In the case of RMSEA, values less than 0.06 and for SRMSEA, less than 0.08 [36].

To obtain evidence of test–retest reliability, the intraclass correlation coefficient (ICC) was estimated with its respective 95% reliability interval, in order to determine the degree of consistency between measurements. In this context, a two-way mixed-effects model was used to achieve measures of “absolute agreement”. Although no consensus was reached regarding the interpretation of this coefficient, some guidelines were established. For the purposes of this study, values higher than 0.75 were considered acceptable reliability values. Values between less than 0.75 and 0.5 were considered moderately reliable and those below 0.5 insufficiently reliable [37].

The internal consistency reliability was obtained through ordinal Cronbach’s alpha and McDonald’s omega [38]. Values higher than 0.7 were considered acceptable for the factor hypothesized.

The above-described processing and subsequent statistical analysis were carried out using Rstudio software, version 1.4.1743–4.

## Results

### Cultural adaptation of OHAT

In general terms, no difficulties were encountered in the translation of the original instrument. Some conceptual terms were modified by consensus, among which were the concepts ‘patchy’, ‘swollen’, ‘rope-like’, and the concept ‘changes’ (second category of the instrument), which was replaced by “signs of possible disease”.

The reverse translation report contemplated a comparison with the original instrument subsequent to the process, together with a valuation of the linguistic equivalence of the translation produced. In this context, a majority of the elements of the OHAT were categorized within a semantic-conceptual equivalence framework, except for the items tongue, gums and tissues and dentures, which were categorized as functional equivalences given the previously reported modifications.

**Fig. 1:**
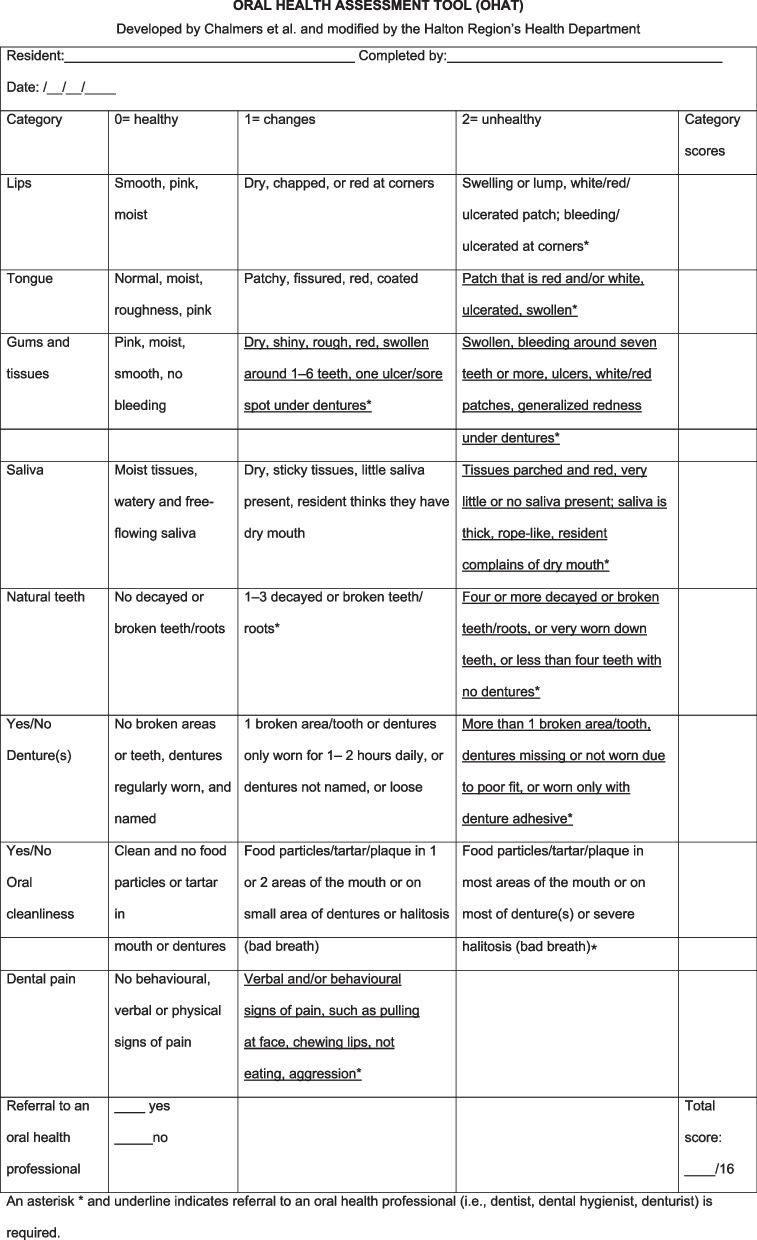
Original version of the OHAT instrument

**Fig. 2:**
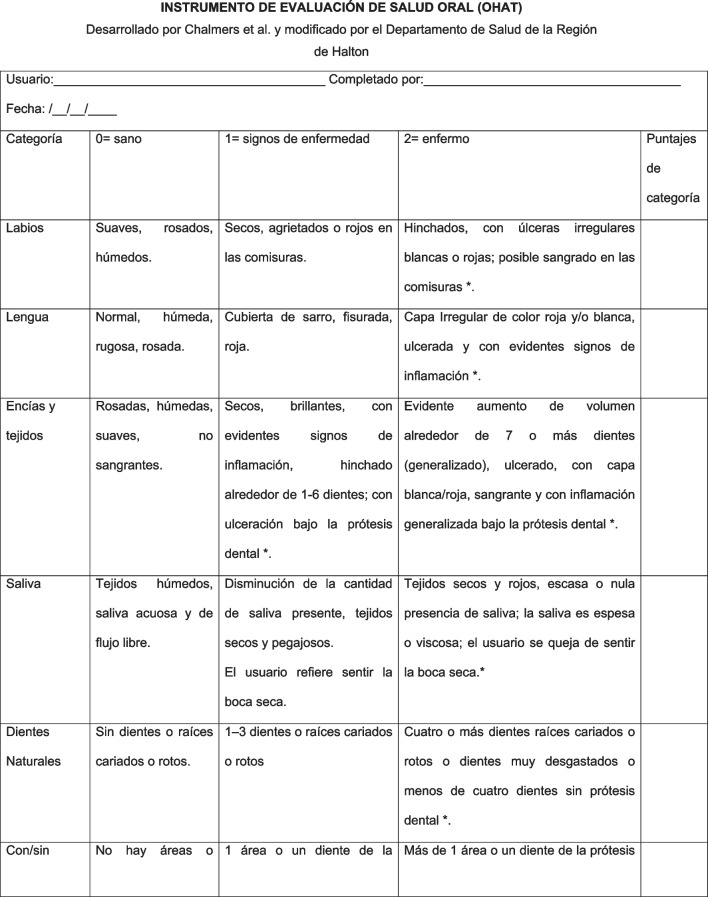

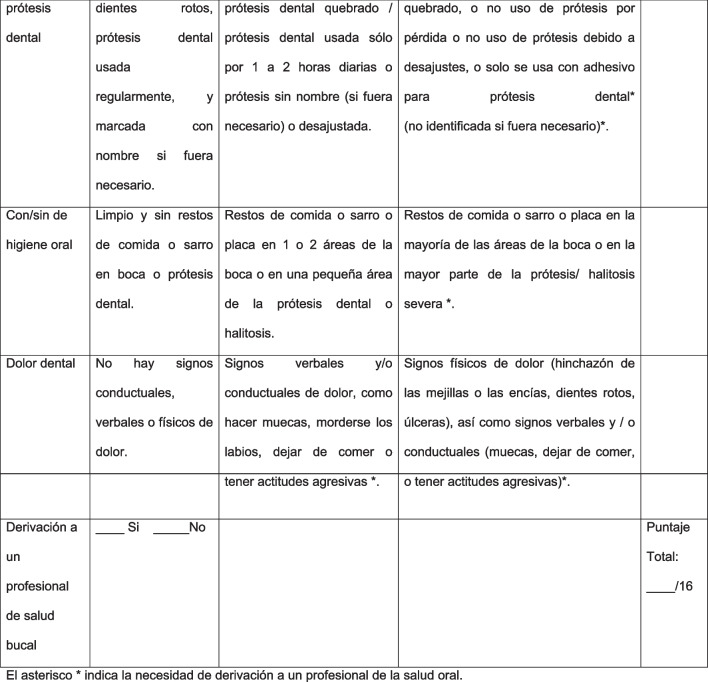
Final version of the OHAT instrument translated and adapted to Chilean Spanish

In the case of dentures, the content of the text was modified at the time of producing the final version of the instrument, where the experts analyzed whether to include “name on dentures” in all categories of the item, because this is not usual practice in Chile in any context. It was decided to maintain the descriptor, but in conditional form (in contexts where it might be necessary). Figures 1 and 2 show the original version of the OHAT and the final consensus version translated into Spanish, respectively. The pre-test of the instrument was undertaken by three selected evaluators, speech-language therapists by profession, experienced in attending to older people for this purpose. Each one was interviewed to get to know their opinion about the general and conceptual comprehensibility of the test and its applicability within the context of speech and language assessment. After this, they were asked to evaluate a total of 30 persons (10 each). Consulted regarding their experience using OHAT, all the professionals stated that the application of the instrument is not difficult in general, the description of the items is clear and leaves no room for doubt, and reported an estimated average duration of 7 min in its application.

### Descriptive analysis

The total initial population consisted of 293 participants. Of these, seven persons were excluded from the study—three for not signing the informed consent form and four because they presented dementia as a base condition, which did not allow them to comprehend or follow simple instructions in the application of the instrument—leaving 286 persons as the total end sample. All the participants fully completed the evaluation. No missing item responses reported. The characteristics of the population participating in the study are described in Table 1.

**Table 1:** Sociodemographic characterization of the study population

Characteristic	*n* (%)
*Gender*	
Female	166 (58)
Male	120 (42)
*Provenance*	
Urban	224 (78.3)
Rural	62 (21.7)
*Type of residence*	
Hospitalized	36 (12.6)
Geriatric residence (State-funded/Private)	59 (20.6)
Residential care for prostrated patients	74 (25.9)
Older persons group	43 (15.0)
Day center	61 (21.3)
Home visit	13 (4.5)
*Dependence level*	
Independent	50 (17.5)
Mildly dependent	134 (46.9)
Moderately dependent	15 (5.2)
Severely dependent	27 (9.4)
Fully dependent	60 (21.0)
*Cognitive performance*	
No deterioration	111 (38.8)
Mild/moderate deterioration	110 (38.5)
Severe deterioration	65 (22.7)

The average age of the participants in the study was 75.01 ± 9.4 years. With regard to a background of pathologies or associated conditions, of the total number of participants 68.5% have arterial hypertension (*n* = 196), 27.3% type 2 diabetes (*n* = 78), 17.8% stroke (*n* = 51), 4.9% concussion (*n* = 14), 5.2% some type of cancer (*n* = 15, more specifically prostate and stomach), 12.6% hypo or hyperthyroidism (*n* = 36), 24.5% hearing loss (*n* = 70), 11.5% present dementia (*n* = 33) and 5.9% have Parkinson’s disease (*n* = 17).

With regard to nutrition, 77.6% (*n* = 222) of the participants eat normally, 11.2% (*n* = 32) eat pureed food and 11.2% are subject to some modification in their nutrition as a result of a medical or speech-language indication (only liquids, only solids, chopped food, etc.). None of the participants were absolutely restricted from ingesting orally, with 5.2% (*n* = 15) currently using a feeding tube to complement their nutrition process and 1% (*n* = 3) fed via gastrostomy.

### Study of psychometric properties

#### Validity

**Fig. 3:**
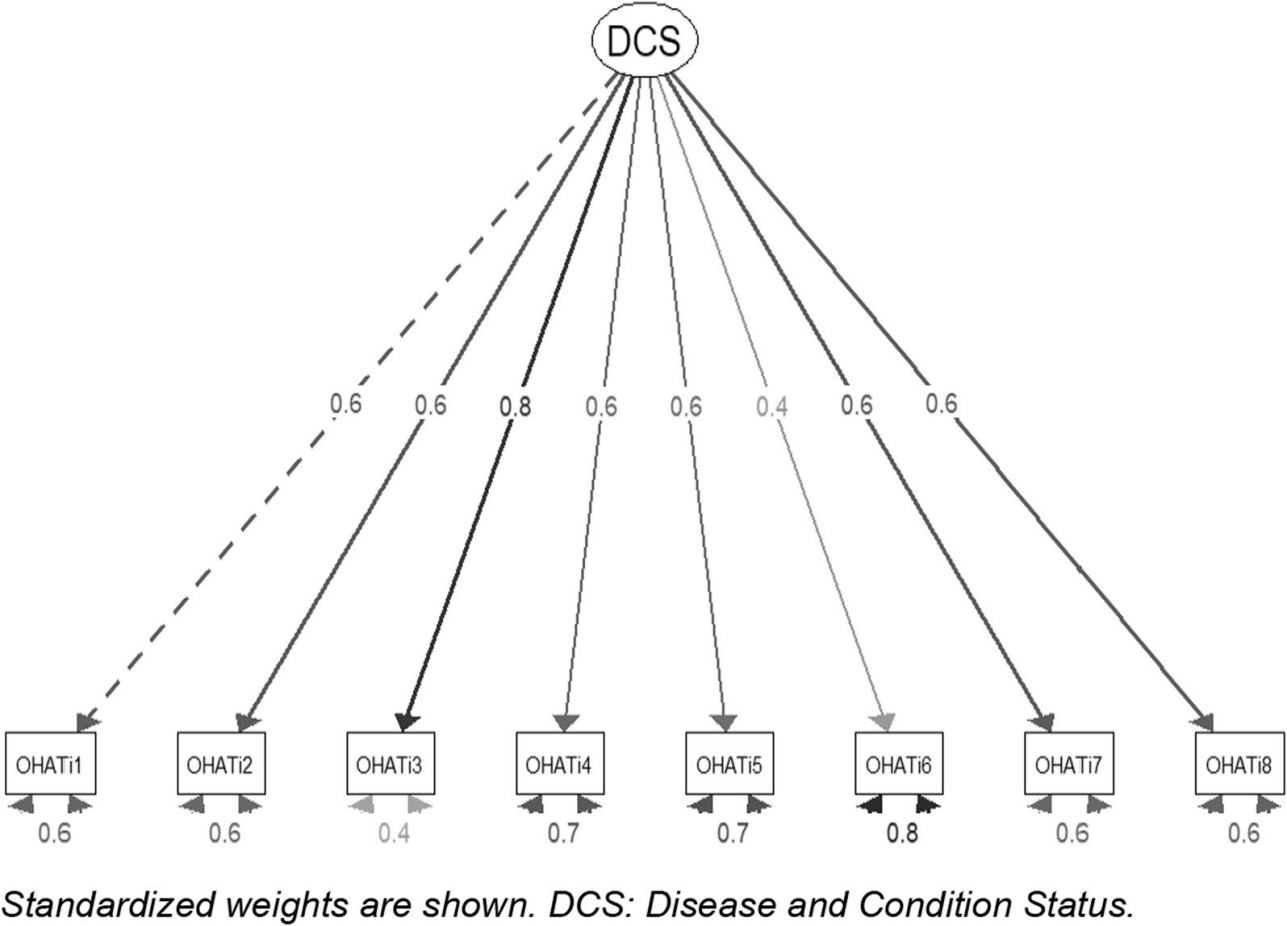
Confirmatory factor analysis on the 8-item OHAT

Figure 3 shows results of the confirmatory factor analysis (CFA) in which the eight items of OHAT loaded on a single latent variable. This model provided a satisfactory adjustment as we hypothesized: DWLS X^2^ = 43.88 (20 degrees of freedom), *p* = 0.002; CFI = 0.98; TLI = 0.97; RMSEA = 0.05 and SRMSEA = 0.07. From these results it is possible to point out that the model fits the data well.

#### Reliability

Internal consistency was carried out calculating the ordinal Cronbach’s alpha (α) coefficient, which resulted in a value of 0.82 for the evaluated dimension. In the same way, the McDonald’s Omega (ω) was 0.87.

Regarding the test–retest reliability A sample of 76 persons was contemplated for the analysis, and was subject to a second valuation that took place 7 days after the first application. The ICC thus obtained was 0.81 (IC95% 0.74–0.87; *p* < 0.001).

## Discussion

The translated and adapted Chilean version of the Oral Health Assessment Tool is presented initially as a tool that is easy to understand and apply. In general terms, the main goal was to achieve conceptual equivalence with the original instrument, safeguarding that any modification made of terms that were difficult to understand or not appropriate for implementation in the Chilean context in general, and more specifically in older adults, was as close as possible to the original meaning. Following this precept, the only concept that differed from the original text was the presence (or absence) of dentures marked with the name of the users, a usual practice in geriatric residences of other countries but currently not applicable to the Chilean reality. Thus, the general valuation of the instrument was not limited solely to the situation of institutionalized older people but to any evaluation context.

In terms of the validity of the instrument and as we hypothesized, the CFA of the OHAT confirmed the one-dimension solution. This is the first study that considered the structural validity of the instrument through confirmatory factor analysis. The factor loadings of the instrument were all greater or equal to 0.4 demonstrating adequate correlation between item and latent variable [39], as well as the previously reported model fit indices. In other validation research such as the Dutch study [23], the number of dimensions extracted did not concur with the dimension hypothesized in the model of the World Dental Federation (FDI) [1], but it is important to stress that the latent variables or constructs to be evaluated tend to differ from culture to culture and thus some variation is to be expected considering the modifications and contexts to which the instrument has been subject [29]. In addition, it is important to mention that these studies used the exploratory factor analysis procedure and that the sample size used was smaller than that recommended by the literature [29, 40].

As mentioned above, the OHAT measures a sub-dimension of oral health (disease and condition status). However, the concept of oral health is complex and multidimensional and not only involves identifying affected structures or functions but also the perceptions of the individuals themselves regarding their health status, or the context that favors or obstructs maintaining their health [1]. In this sense the OHAT is an instrument whose clinical usefulness lies in the observation of structures or consequences in oral functioning that increase risk or detect possible alterations that can lead to disease as a more objective measure than just self-perception or self-report. Consequently, the results obtained in the present research are sufficient to consider that the OHAT has structural validity.

In terms of internal consistency reliability, the reported ordinal Cronbach’s alpha and Mcdonald’s Omega coefficient were good (α = 0.82; ω = 0.87) for the entire one-dimension instrument, similar results than obtained by Kuwamura et al. [41] but differing from the values obtained by Mello et al. [26], who evaluated the internal consistency of the instrument applied by a group of nurses and dentists to 50 older people, obtaining alpha values considered to be low. In this respect, the following should be considered: first of all, it is important to note the definition of this measure, which is frequently considered to refer to the degree in which all elements of a test or instrument measure the same attribute or dimension [42]. In this sense, according to Bonett [43] and Charter [44], sample sizes of more than 250 subjects are required to obtain appropriate and precise values with this coefficient, both for determining the coefficient of the instrument and to validate any comparisons. In this study, the calculation of the coefficient was carried out in compliance with this condition.

In the same vein, another objective of the study was to determine the test–retest reliability or reproducibility of the instrument. This provides an indicator of the stability of the test measurements as a function of a specific interval between two evaluations taken in different time periods [45], which is also of clinical interest. In this study the intraclass correlation coefficient value was 0.81 (IC95% 0.74 – 0.87), considered to be good. Similar values were obtained in the original validation [17] of the OHAT and the English validation directed at evaluators who were speech-language therapists by profession [18].

With regard to the limitations of this study, the aspect of representativeness is mentioned. Although the calculation of the sample size is based on the number of measures per item, it is also necessary to safeguard that, from an epidemiological point of view, there are sufficient contexts available to encompass greater variability. In this sense, access to the population of older people receiving home care (*n* = 13) was lower, and therefore it is likely that such variability was not fully covered.

Another limitation has to do with the process itself. International guidelines on the validation of instruments contemplate other types of validation that are useful when considering whether or not an instrument is valid (and also reliable) [46, 47]. In this sense, it would be useful to develop other studies that would make it possible to compare performance in terms of detecting the OHAT using a previously established criterion (such as, for example, the considered opinion or valuation of a professional dentist), its concurrent and discriminant validity, or likewise its predictive validity. From a methodological viewpoint, a prospective (cohort) study would be useful to test these hypotheses. Notwithstanding the above, previous systematic reviews have reported that for general geriatric assessment, screening or triage carried out by non-dentist professionals, the OHAT is suggested [48]. In this sense, one of the strengths of this research is that it contributes with antecedents regarding the construct to be assessed at the time of evaluation, adding to the existing evidence of content and criterion validity [48].

Although there is evidence available regarding the clinical usefulness of the OHAT in other contexts, especially in terms of its application by speech-language therapists and nurses, no certainty exists regarding the feasibility of its use by the latter professionals given that the training and application of this study was carried out solely by the former. It would be a mistake to assume that the formative processes or conceptual contents of nurses are identical to their similar counterparts in other countries or contexts. It is therefore considered relevant to undertake additional research efforts that will evaluate the feasibility of the use of the OHAT by these evaluators.

Similarly, given variable conditions for assessment, it is necessary to consider future validation studies contemplating more assessment contexts. Currently, the resources of tele-assessment or tele-care associated with the virtualization of oral health care are of interest and growing acceptability given the evidence of its benefits, especially in terms of promotion and prevention related to access to dental or oral health professionals (shortening distances, facilitating access to care) [49, 50], so, having an instrument such as the OHAT adapted for remote application could be an interesting projection regarding these new trends.

## Conclusions

In light of the foregoing, it is considered that the Oral Health Assessment Tool has structural validity and possesses adequate properties of internal consistency and test–retest reliability for the population under study. Its orientation is initially clinical, aimed at favoring the detection of possible oral health problems in Chilean older people and referring them in a timely fashion to a professional dentist for their optimal care. The information provided could be useful also in possible actions of health promotion and disease prevention, not only directed at older adults but also their caretakers and treatment team, and could have an impact on indicators of oral health and particularly in the quality of life of older people. In turn, it is considered as an alternative or complement to the valuation of speech and language structures, and even the valuation of the swallowing process by the speech-language therapists, who could use the instrument in future lines of research to determine its contribution to the diagnostic or decision-making process regarding the treatment of swallowing disorders in the elderly.

Finally, it is important to undertake further studies of different types of validity and reliability in order to collect more information about the psychometric properties of the instrument and extend or project its usefulness into the future. By the same token, it is useful to stress that no instrument is definitive in terms of its psychometric properties, thus making it advisable to review and enrich the instrument over time in order to improve its characteristics.

## Data Availability

The datasets generated during and analyzed during the current study are not publicly available due to data protection regulations and ethical concerns but are available from the corresponding author on reasonable request.

## Abbreviations

CFI: Comparative fit index
CFA: Confirmatory factor analysis
DWLS: Diagonally weighted least squares
DCS: Disease and condition status
FDI: World dental federation
GOHAI: Geriatric oral health assessment index
ICC: Intraclass correlation coefficient
OHAT: Oral Health Assessment Tool
OHIP: Oral health impact profile
RMSEA: Root mean square error of approximation
SPMSQ: Short portable mental state questionnaire
SRMSEA: Standardized root mean square error of approximation
TLI: Tucker-Lewis index
WHO: World health organization
